# Growth, Feeding Tolerance and Metabolism in Extreme Preterm Infants under an Exclusive Human Milk Diet

**DOI:** 10.3390/nu11071443

**Published:** 2019-06-26

**Authors:** Fabian Eibensteiner, Lorenz Auer-Hackenberg, Bernd Jilma, Margarita Thanhaeuser, Martin Wald, Nadja Haiden

**Affiliations:** 1 Department of Pediatrics, Division of Neonatology, Pediatric Intensive Care and Neuropediatrics, Medical University of Vienna, 1090 Vienna, Austria; 2 Department of Pediatrics, Division of Neonatology, Paracelsus Medical University, 5020 Salzburg, Austria; 3 Department of Clinical Pharmacology, Medical University of Vienna, 1090 Vienna, Austria

**Keywords:** ELBW-infants, exclusive human milk diet, growth velocity, fortification of human milk, feeding tolerance

## Abstract

Background: For preterm infants, human milk (HM) has to be fortified to cover their enhanced nutritional requirements and establish adequate growth. Most HM fortifiers are based on bovine protein sources (BMF). An HM fortifier based on human protein sources (HMF) has become available in the last few years. The aim of this study is to investigate the impact of an HMF versus BMF on growth in extremely low birth weight (ELBW, <1000 g) infants. Methods: This was a retrospective, controlled, multicenter cohort study in infants with a birthweight below 1000 g. The HMF group received an exclusive HM diet up to 32+0 weeks of gestation and was changed to BMF afterwards. The BMF group received HM+BMF from fortifier introduction up to 37+0 weeks. Results: 192 extremely low birth weight (ELBW)-infants were included (HMF *n* = 96, BMF *n* = 96) in the study. After the introduction of fortification, growth velocity up to 32+0 weeks was significantly lower in the HMF group (16.5 g/kg/day) in comparison to the BMF group (18.9 g/kg/day, *p* = 0.009) whereas all other growth parameters did not differ from birth up to 37+0 weeks. Necrotizing enterocolitis (NEC) incidence was 10% in the HMF and 8% in the BMF group. Conclusion: Results from this study do not support the superiority of HFM over BMF in ELBW infants.

## 1. Introduction

Preterm infants need an enhanced intake of nutrients to achieve intrauterine growth rates during their early postnatal life [1,2,3,4]. Human milk (HM) is the gold standard of care for all newborn infants, and can be administered as either birth mother or donor mother milk [5,6]. However, human milk is designed for term infants and does not provide enough macro- and micronutrients to cover the nutritional requirements of preterm infants [4,7,8,9]. These requirements may be further increased by a complicated postnatal course in the intensive care unit, as well as diseases associated with prematurity such as necrotizing enterocolitis (NEC) or bronchopulmonary dysplasia (BPD) [10]. Therefore, human milk has to be fortified with multicomponent fortifiers containing extra protein, calories, electrolytes and vitamins. Fortification of human milk prevents postnatal growth failure and contributes to a normal long-term neurodevelopmental outcome [7,8]. Most of the commercially available human milk fortifiers are based on bovine protein sources. A human milk fortifier based on human protein sources has become available over the last few years. The first clinical studies provide additional advantages when compared to human milk feedings with bovine based fortifiers, especially in very low birth weight infants (VLBW, <1500 g birthweight). An exclusive human milk (EHM) diet might be associated with a lower NEC incidence [11], a better feeding tolerance [12], a shorter time until full enteral feedings [12] leading to a shorter duration of hospital stay, and lower overall costs in comparison to bovine based human milk fortifiers (BMF) and formula [12]. However, the composition of the human milk based human milk fortifier (HMF) differs to the composition of bovine products with a lower carbohydrate content and a higher fat content (Protein-energy-ratio: HM 2.59 g/100kcal, HMF 3.37 g/100kcal, BMF 3.42 g/100kcal), which might affect growth and weight gain of preterm infants. So far, there is only limited data available on growth and weight gain of very immature infants with a birthweight below 1000 g under an exclusive human milk diet. We hypothesize that there may be a difference in growth velocity between extremely low birth weight infants (ELBW-infants, <1000 g birthweight) fed with HMF in comparison to infants fed with BMF. Furthermore, we determined the effect of an exclusive milk diet on other parameters of growth, time to full enteral feedings, fortifier tolerance, glucose and fat metabolism, and morbidities such as NEC.

## 2. Materials and Methods

The retrospective observational multicenter study was conducted at two university tertiary care centers in Vienna and Salzburg. Two different fortification periods were compared: The exclusive human milk diet group received HM fortified with a human milk based fortifier (Prolact+6 H2MF^®^ Prolacta, City of Industry, California = HMF group, Center 1: 12/2015-5/2016, Center 2: 1/2016-9/2018), the control group received HM fortified with a bovine based fortifier (Aptamil FMS^®^ 4.4% Milupa, Puch, Hallein, Austria or Beba FM85 4% Nestlé Nutrition GmbH, Frankfurt am Main, Germany = BMF group, Center 1: 5/2012-11/2015 and 7/2016-11/2016, Center 2: 7/2013-12/2015). The HMF group was matched 1:1 for birthweight (within 100 g) and gestational age at birth (within 7 days) with the bovine control group. Inclusion criteria were a birthweight <1000 g and initiation of human milk fortification. Exclusion criteria were congenital heart disease, major congenital birth defects or major inborn error of metabolism. The study was approved by the ethics committee of the Medical University of Vienna (1464/2016) and registered at clinicaltrials.gov (NCT03886844). All the parents were informed that their child is admitted to a university hospital and that data might be used for research purposes.

### 2.1. Standardized Feeding Regimen and Study Groups

**Figure 1:**
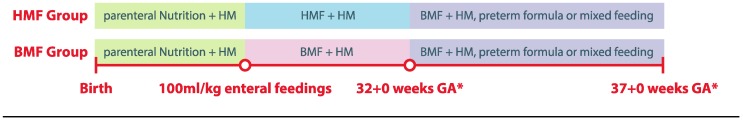
Feeding regimen of the study groups. *) GA = gestational age corrected for prematurity; HM = Human milk, BMF = bovine based fortifier, HMF = human milk-based fortifier.

Both groups and centers adhered to a standardized parenteral and enteral feeding regimen: Parenteral nutrition as recommended by ESPGHAN (European Society for Pediatric Gastroenterology, Hepatology and Nutrition) guidelines [13] was started immediately after birth and continued until enteral intake was at least 140 mL/kg/d. Parenteral nutrition was administered via a peripherally inserted central catheter (PICC)-line, which was inserted in the first 36 h of life and removed as soon as the infant was on full enteral feedings. Minimal enteral feedings (1 mL every 2–4 h) were started after the first 3–6 h or at the latest within 24 h of life. Colostrum of the premature infant’s mother or donor human milk was exclusively used for the introduction of enteral nutrition and up to at least 32+0 weeks [14]. A daily increase of enteral feeding volume by 20 mL/kg/d was aimed for [14,15]. Fortification of HM was started as soon as an infant tolerated an enteral intake of 100 mL/kg. Therefore, infants were included in the study once an enteral feeding volume of 100 mL/kg/d was reached and fortification of human milk was started (Figure 1).

#### 2.1.1. HMF Group

Human milk was fortified in the ratio of 70 mL breast milk to 30 mL liquid human milk-based fortifier (HMF). At 32+0, corrected for prematurity, HMF was replaced stepwise by bovine based fortifier (BMF) over a period of 7 days. During the first two days, two of eight feedings were supplemented with BMF instead of HMF. For the next four days, four of eight feedings were replaced and on day seven all feedings were fortified with BMF. Subsequently fortified human milk was used whenever possible up to 37+0, which was the end of the study period. If not enough HM was available, infants received mixed feedings or preterm formula (Nestlé BEBA^®^ Frühgeborenennahrung Stufe 1, Nestlé Nutrition GmbH, Frankfurt am Main, Germany or Aptamil Prematil^®^ HA, Milupa Nutricia GmbH, Frankfurt am Main, Germany).

#### 2.1.2. BMF Group

HM fortified with powdered bovine based fortifier (BMF) was used for fortification up to a minimum of 32+0 weeks. Subsequently fortified human milk was fed whenever possible up to 37+0 which was the end of the study period. If not enough HM was available, infants received mixed feeding or preterm formula (Nestlé BEBA^®^ Frühgeborenennahrung Stufe 1, Nestlé Nutrition GmbH, Frankfurt am Main, Germany; or Aptamil Prematil^®^ HA, Milupa Nutricia GmbH, Frankfurt am Main, Germany).

Macronutrient content and macronutrient-energy-ratios of HM, HM+HMF and HM+BMF are given in Table 1. Both groups received 6 mg/kg/d iron supplementation (Ferrum Hausman^®^ [iron-III-polymaltose-complex] or Aktiferrin^®^ [iron-II-sulfate] [16]) starting on the 15th day of life until discharge. The observation period ended at a gestational age of 37+0 weeks or discharge, whichever came first.

### 2.2. Outcome Measures

All data were obtained by reviewing the electronic patient record forms (Center 1: ICIP Philips, Austria; SAP3^®^ SAP, Austria; catoPAN™ Becton Dickson, Austria; Center 2: MetaVision Version 5.46.42 iMDsoft BV The Netherlands Schipholweg 86, 2316 XD Leiden; Headq: Ltd. Kiryat Atidim #4, POB 58178, Tel Aviv 6158; KIS Orbis SP 08.04.31).

#### 2.2.1. Parameters of Growth

Growth velocity (g/kg/day) was calculated according to the exponential method described by Patel et al. [17]:

$$GV=\frac{1000*\ln\frac{W_{n}}{W_{1}}}{D_{n}}$$

*GV…growth velocity, W_n_…weight at time point of interest, W_1_…weight at birth, D_n_…day of life at time point of interest*

The formula was adapted for the growth velocity (g/kg/day) calculation from fortifier introduction to 32+0 weeks of gestation as follows:

$$GV=\frac{1000*\ln\frac{W_{n}}{W_{1}}}{D_{n}-D_{1}}$$

*GV…growth velocity, W_n_…weight at time point of interest, W_1_…weight at fortifier introduction, D_n_…day of life at time point of interest, D_1_…day of life at fortifier introduction*

Z-scores of weight, length, and head circumference were calculated using Fenton growth charts [18]. small for gestational age (SGA) was defined as birthweight <10th percentile.

#### 2.2.2. Time to Full Enteral Feeding and Feeding Tolerance

Time to full enteral feeding was defined as days until a minimum enteral feeding volume of 140 mL/kg/d was achieved. Partial parenteral nutrition was stopped and the central line was usually removed in parallel, unless the infant was still receiving antibiotics. In this case the central line was removed on completion of antimicrobial therapy. Feeding intolerance was defined as gastric residuals and vomiting and/or abdominal distension and/or discontinuation of fortification. Gastric residuals were defined as >50% of previous feeding volume [19]. All of these parameters, as well as data on stool changes (obstipation, liquid stools, change of color or change of frequency), spitting, and vomiting were obtained during the first three days after fortifier introduction and during fortifier transition.

#### 2.2.3. Parameters of Glucose and Fat Metabolism

Serum blood glucose was measured whenever a routine lab was done, for example, blood gas analysis or sepsis workup. Serum triglycerides were measured at least once a week. Hypoglycemia was defined as glucose values lower than 45 mg/dL and hypertriglyceridemia as triglyceride values higher than 200 mg/dL. In the present study, values where only included under the following conditions: (1) full enteral feedings (2) fortification of human milk (3) maximal gestational age 32+0 weeks respectively.

#### 2.2.4. Morbidity

Intraventricular hemorrhage (IVH) was defined according to Papile [20], necrotizing enterocolitis (NEC) according to Bell [21], bronchopulmonary dysplasia (BPD) was defined as a FiO_2_ of >0.21 at a gestational age of 36+0 weeks [22], sepsis as at least one positive blood culture, periventricular leukomalacia (PVL) according to de Vries [23], retinopathy of prematurity (ROP) according to The International Committee for the Classification of ROP [24], focal intestinal perforation (FIP) according to Donahue [25]. A hemodynamically significant persistent ductus arteriosus (PDA) was defined as requiring treatment with Ibuprofen or surgical closure.

### 2.3. Statistical Analysis

Based on available data, a sample size calculation was only possible for growth velocity from birth up to 37+0 weeks of gestation: 10% was considered as a minimally relevant effect, and a weighted mean of 13.35 and a weighted SD of 2.3 were derived for the HFM group from previous publications [16,26]. Thus, a sample size of 96 patients per group was sufficient to detect this effect with 90% power at a two-sided significance level of 1%. The study’s primary outcome parameter was growth velocity (g/kg/d) from birth up to 37+0 weeks of gestation. The most sensitive endpoint, however, was growth velocity from fortifier introduction up to 32+0 weeks of gestation, because it was not influenced by factors occurring before or after the use of fortifier. Other endpoints included growth velocity (g/kg/d) from birth up to 32+0 weeks of gestation, length and head circumference increase (cm/week) from birth until 32+0 and 37+0 weeks of gestation. To adjust for the multiplicity of those five endpoints, a two-tailed *p*-value of <0.01 was considered significant. Exploratory and safety endpoints included time to full enteral feedings (140 mL/kg/d), days on central line, NEC-incidence (at least Bell IIa), and the incidence of hypoglycemia and hypertriglyceridemia from full enteral feedings with fortification up to 32+0 weeks of gestation. Statistical analysis was performed using IBM SPSS V.24.0.0.0 2016 (SPSS, Inc., Chicago, IL, USA) and R Core Team (2018; R: https://www.R-project.org/). Categorical data were summarized using absolute and relative frequencies. Continuous data were summarized using medians, means, interquartile ranges (IQR) and non-parametric 95% confidence intervals (CI95%). Differences in baseline characteristics, morbidity, and mortality between groups were computed using Mann-Whitney U test and Chi-Squared test (or Fishers Exact test) as appropriate. Differences in primary and secondary outcome parameters between groups and study centers were computed using Mann-Whitney U test and Chi-Squared test (or Fishers Exact test) as appropriate.

## 3. Results

In total 192 ELBW-infants were included in our final analysis: 96 infants in the HMF group and 96 infants in the BMF group. Center 1 contributed a total of 82 infants (*n* = 41 HMF group, *n* = 41 BMF group), center 2 contributed a total of 110 infants (*n* = 55 HMF group, *n* = 55 BMF group) for data analysis.

### 3.1. Baseline Characteristics, Morbidity and Mortality

A summary of baseline characteristics is given in Table 2. Groups were balanced with regards to birthweight and gestational age: Median gestational age in the HMF group was 26+1 weeks (IQR 24+6, 27+1) versus 25+6 (IQR 24+5, 27+3) weeks in the BMF group. Median birthweight was 752 g (IQR 659, 893 g) and 773 g (IQR 650, 890 g) respectively. There were no statistically significant differences between groups or centers, except for the number of premature rupture of membranes (PROM). The incidence of PROM was significantly higher in the HMF group (40%) than in the BMF group (23%).

### 3.2. Primary and Secondary Outcomes

#### 3.2.1. Growth

**Figure 2:**
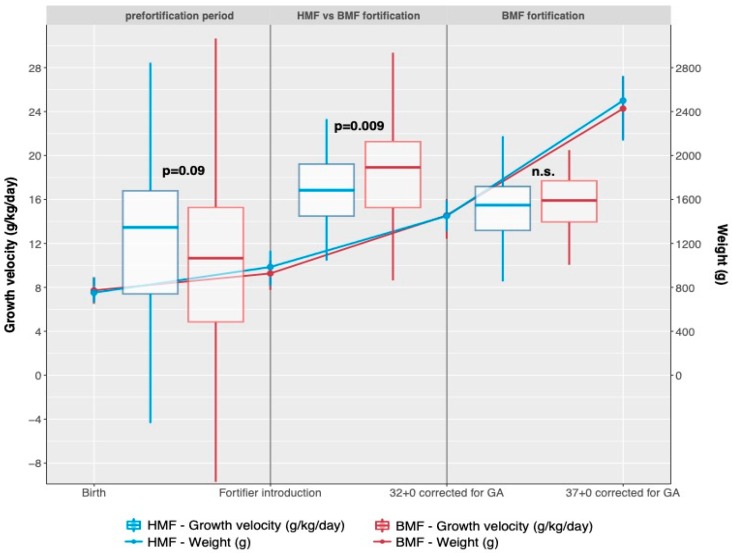
Comparison of absolute weight and growth velocities between groups.

Growth velocity (g/kg/day), length increase (cm/week) and head circumference increase (cm/week) up to 32+0 and 37+0 weeks of gestation did not differ significantly between the HMF and BMF groups (Table 3). Furthermore, there were no statistically significant differences in the absolute values of weight, length or head circumference at 32+0 and 37+0 weeks of gestation. However, growth velocity differed significantly in the period from the introduction of fortifier up to 32+0 weeks of gestation: 16.5 g/kg/d (= median, CI95% 6.5–33.7 g/kg/d) in the HMF group versus 18.9 g/kg/d (= median, CI95% 10.2–31.8 g/kg/d) in the BMF group (*p* = 0.009) indicating that during this time period the HMF group grew slower than the BMF group (Table 3, Figure 2).

In contrast, growth velocity before the introduction of fortifier (from birth to introduction of fortifier) was 3 g/kg/d higher in the HMF group (median 13.5 g/kg/d, CI95% 6.9–23.8 g/kg/d) than in the BMF group (median 10.7 g/kg/d, CI9% 6.7–28.3 g/kg/d) with a trend towards significance (*p* = 0.09, Table 3). This difference could not be explained by the age of the infants at fortifier introduction or by duration of different fortifier regimen. In the HMF group, fortification of breast milk was started on the 17th day of life (= median, IQR 12, 24) and at a gestational age of 28+4 weeks (= median, IQR 27+5, 30+3). In the BMF group, fortification was started on the 15th day of life (= median, IQR 11, 21; *p* = n.s.) and at a gestational age of 28+6 weeks (= median, IQR 27+2 to 29+6; *p* = n.s.). Time of fortification from fortifier introduction up to 32+0 days was 20 days (= median, IQR 8, 28 days) in the HMF group and 19 days (= median, IQR 10, 30 days) in the BMF group (*p* = n.s).

The total number of fortifier days until 37+0 was 48 days (= median, IQR 35, 59 days) in the HMF and 45 days (= median, IQR 31, 57 days) in the BMF group (*p* = n.s.).

#### 3.2.2. Time to full enteral feedings

Time to full enteral feedings, duration of parenteral nutrition and central line days were significantly longer in the HMF group than in the BMF group (Table 4).

However, in both groups the central line was removed two days after termination of parenteral nutrition. This was mainly due to 24-h administration of antibiotics. After the completion of treatment, the central line was removed.

Up to 32+0 weeks, all infants received fortified HM according to their group exclusively. After 32+0 up to 37+0 weeks infants were fed with fortified HM whenever possible. If not enough HM was available, infants received mixed feedings or preterm formula (Table 4).

#### 3.2.3. Fortifier Introduction and Transition from HMF to BMF Fortification

**Figure 3:**
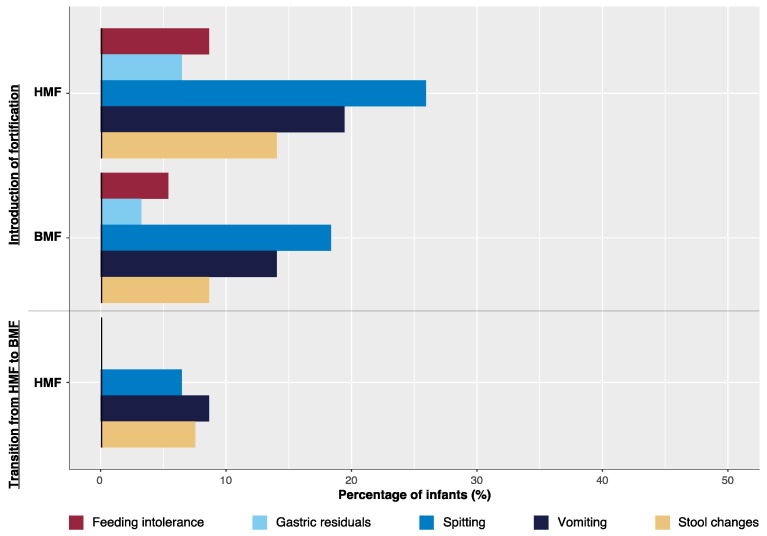
Differences in gastrointestinal tolerance during fortifier introduction and fortifier transition between groups.

During fortifier introduction 8% (HMF group) and 5% (BMF group) of infants showed signs of feeding intolerance (Figure 3). Gastric residuals alone occurred in 6% (HMF group) and 3% (BMF group) of infants. Changes in stool pattern occurred in 13% (HMF group) and 8% (BMF group), spitting in 24% (HMF group) and 18% (BMF group), and vomiting in 18% (HMF group) and 14% (BMF group) of infants during the introduction of fortification (Figure 3). Symptoms did not differ significantly between groups.

Up to 32+0 weeks fortification was briefly interrupted in 23% (*n* = 23) of the HMF group and 19% (*n* = 18) of infants BMF group (*p* = n.s., Table 5). Reasons for the discontinuation of fortifier application were feeding intolerance with suspicion of NEC and/or cardiorespiratory instability due to sepsis.

#### 3.2.4. Transition of Fortifier

In accordance with our study protocol, infants in the HMF group were swapped from HMF to BMF fortifier at 32+0 weeks of gestation. Fortifier transition was conducted on the 44th day of life (= median, IQR 34,54) at a corrected gestational age of 32+2 weeks (= median, IQR 31+3,33+2), and a weight of 1448 g (= median, IQR 1345,1589 g).

Adherence to a transition protocol of seven days was 41% (*n* = 39), 59% of the infants had a shorter transition of four days (= median; IQR 3,6). During this transition period in 20% (*n* = 20) of the infants one or more of the following occurred: Stool changes in 5% (*n* = 5), spitting in 6% (*n* = 6), and vomiting in 8% (*n* = 8). None of infants showed signs of feeding intolerance. Transition from HMF to BMF was interrupted in 5% (*n* = 5) of infants due to suspicion of NEC, surgical interventions (each case of: orchidopexia, implementation of a ventriculoperitoneal shunt, stoma reconstruction), and insufficient weight gain.

Data on fortifier introduction and transition are displayed in Figure 3.

#### 3.2.5. Parameters of Glucose and Fat Metabolism

The incidence of hypertriglyceridemia during full enteral feeding with fortification up to 32+0 weeks of gestation was 4% (*n* = 4) in the HMF and 5% (*n* = 5) in the BMF group respectively (*p* = n.s). Hypoglycemia occurred in 6% (*n* = 6) in the HMF and 11% (*n* = 11) in the BMF group, which was not statistically significant (*p* = n.s.)

#### 3.2.6. Morbidity and Mortality

Data on morbidity and mortality are given in Table 6. There were no statistically significant differences in major morbidities and mortality between the HMF and BMF group. Incidence of NEC was 10% in the HMF group and 8% in the BMF group, with a median onset on the 20th (IQR 16–24) and 22nd (IQR 15-34) day of life respectively. At NEC onset 80% (*n* = 8) of the infants in the HMF group and 62.5% (*n* = 5) in the BMF group received fortified HM – the other 20% (*n* = 2; HMF group) and 37.5% (*n* = 3; BMF group) infants with NEC received unfortified HM with or without parenteral nutrition. FIP occurred in 5% of the infants in the HMF group (onset day 9; = Median; IQR 611) and in 5% of the infants in the BMF group (onset day 12, = median; IQR 11,14) respectively.

## 4. Discussion

In a retrospective observational, controlled, multicenter cohort study, we investigated the effect an exclusive human milk diet up to the 32+0 weeks of gestation had on growth velocity, time to full enteral feedings, feeding tolerance, and major morbidities in ELBW-infants when compared to BMF. Growth velocity as well as other growth parameters up to 32+0 and 37+0 weeks of gestation did not differ between the study groups. Growth velocity in ELBW-infants was significantly slower under the HMF regimen (16.5 g/kg/day) from the introduction of fortifier up to 32+0 weeks of gestation in comparison to a BMF regimen (18.9 g/kg/day). In the HMF group, time to full enteral feedings was six days longer than in the BMF group (*p* = 0.0018). Both groups showed mild signs of feeding intolerance such as spitting, vomiting, or gastric residuals during fortifier introduction and fortifier transition—however, these findings did not tend to be clinically relevant. There was no difference in NEC, other major morbidities, and mortality between groups. In addition, the incidence of hypoglycemia and hypertriglyceridemia did not differ between the two feeding regimens.

### 4.1. Growth

Fortification of HM is strongly recommended for preterm infants to achieve intrauterine growth rates, avoid postnatal growth restriction, and facilitate an appropriate neurodevelopmental outcome [1,5]. The optimal extrauterine growth velocity for preterm infants is not yet fully clear, but most experts and large pediatric societies, such as the ESPGHAN, recommend a weight gain of 17–20 g/kg per day in very low birth weight infants after the initial postnatal nadir of weight loss [28]. The new human milk-based fortifier has a different composition from bovine products with a higher protein and fat content and a lower carbohydrate content (Table 1), and might be helpful in achieving the anticipated 17–20 g in the early postnatal period. One of the first studies was promising and reported growth velocities of 24.8 g/kg/d in infants with a birthweight <1250 g when HMF was started at an enteral intake of 60 mL/kg/d [29]. However, this high growth velocity rate was not confirmed by other studies (Table 7).

In this study, we introduced the new fortifier in two tertiary care centers in Austria. Additionally, this was the first time the fortifier was provided to the smallest preterm infants with a birthweight below 1000 g during the most vulnerable timespan. This is the time from the introduction of fortification at a minimum of 100 mL/kg enteral intake up to an age of 32+0 weeks. During this time period in the HMF group growth velocity was 16.5 g/kg/d, which was significantly lower than in the BMF group (18.9 g/kg/d), whereas day of life at introduction and days on fortifier were comparable (Figure 2). Overall growth velocity from birth to 32+0 and 37+0 weeks, length increase, and increase in head circumference did not differ between groups (Table 3), but was lower (15.2 g/kg/d in HMF and 15.6 g/kg/d in the BMF group) than the recommended 17–20 g/kg/d. Based on the available data, a sample size calculation was only possible for growth velocity from birth up to 37+0 weeks of gestation, which was the primary outcome of the study. However, growth velocity from fortifier introduction to 32+0 weeks can be considered more sensitive in this study setting.

A protein-energy-ratio of 3.4 g/100kcal is needed to establish adequate growth in preterm infants at a gestational age of 26 to 30 weeks [14]. Human milk has a protein-energy-ratio of 2.59 g/100 kcal, in comparison to 3.37 g/100 kcal of HM fortified with a HMF and 3.42 g/100 kcal of HM fortified with a BMF, indicating that both fortifiers had optimal protein-energy-ratios to establish adequate growth (Table 1). However, the energy content of the HMF regimen is characterized by a high lipid content (5.5 g/100 mL) and a low carbohydrate content (7.63 g/100 mL). In contrast, the BMF consists of a high carbohydrate content (10 g/100 mL) and a low lipid content (3.5 g/100 mL). In preterm infants approximately 20–30% of enteral fed lipids are excreted via stool due to an insufficient intestinal fat digestion and absorption, mainly caused by low gastric, hepatic and pancreatic enzyme secretion (especially lipase and bile salt) [14,30]. It is likely that in this group of very preterm infants the energy provision from fat is impaired by a lower fat digestion and absorption. However, a minimum of 30–40 kcal per 1 g amino acids is usually recommended to guarantee amino acid utilization [31]. Carbohydrates might be a more readily available source of energy, as carbohydrate digestion is hardly affected in preterm infants. The ESPGHAN recommends a minimal carbohydrate-energy-ratio of 10.5 g/100 kcal for adequate enteral intake. The HM+HMF mixture only provides a carbohydrate-energy-ratio of 8.4 g/100 kcal, which is lower than recommended. In contrast, the HM+BMF mixture provides a carbohydrate-energy-ratio of 11.4 g/100 kcal. Therefore, we hypothesize that the less favorable carbohydrate-energy-ratio might be the reason for the significantly lower growth rate from fortifier introduction up to 32+0 weeks in infants of the HMF group.

Other authors also reported on slower velocities under an exclusive HM diet in comparison to one with bovine fortification. Additionally, the recommended growth velocity of 17–20 g/kg/d could not be achieved in most of the published studies (Table 7).

### 4.2. Time to Full Enteral Feedings, Fortifier Introduction and Transition

Time to full enteral feedings defined as a minimal enteral intake of 140 mL/kg lasted six days longer in the HMF (26 days) than in the BMF group (20 days, *p* = 0.0018, Table 4). This is in line with data from ELBW-infants showing that time to full enteral feedings in the HMF group was achieved 2.5 days later than in the BMF group (29 days in HMF versus 26.5 days in the BMF group, *p* = n.s.) [26]. In contrast, studies in larger, more mature preterm infants report an additional 9.25 [12] and 6.8 [29] days required to reach full enteral feeding when infants received BMF instead of HMF, but differences in birthweight and gestational age influence comparability of data. Other studies in more mature preterm infants could not find differences in time to full enteral feedings between human and bovine fortification regimens. They report time periods of 22 days [11], 24.6 days [32] or 25.3 days [33] under an exclusive human milk diet, which is comparable to our results, although we focused on smaller and younger preterm infants. Data on feeding tolerance are also conflicting: there was no difference in feeding tolerance, gastric residuals, or feeding interruption in the present study and the overall number of infants with feeding problems was low. This is in line with the data published by O’Connor [27] who collected data for the whole fortification period (Table 5). Again, other authors refer on a better feeding tolerance in more mature infants with exclusive human milk diet with only 6% feeding withheld in the group of HMF (34% in the BMF group, *p* = 0.001) [12].

These data indicate that feeding tolerance is strongly related to the degree of prematurity but not to the type of human milk fortification.

### 4.3. Morbidity and Mortality

An exclusive human milk (EHM) diet was found to be associated with a lower incidence of NEC^11 32^, BPD, ROP, late-onset sepsis and mortality [34]. Conversely in the present study we did not find any differences in the incidence of major morbidities (NEC, FIP, BPD, ROP, Sepsis, IVH, PDA) and mortality. NEC incidence in the HMF group was 10% and was 8% in the BMF group. This is higher than in the Vermont Oxford Network (VON) where the overall NEC incidence in infants between 22–29 weeks is reported as 6% and the NEC incidence in survivors is reported as 3% [35]. However, most of the studies investigating an exclusive human milk diet showed a similar NEC incidence of 3–10% in their human milk groups [16,26,29,36]. In these studies, the NEC rate in the BMF groups was sometimes more than twofold higher (11% and 16% [11,26]) than in our BMF group, although some of the studies were not restricted to infants with a birthweight below 1000 g but rather included more mature preterm infants. It is likely that NEC rates of 16–21% are not only related to the type of fortification of human milk [11] or formula [32], but also to other factors such as the lack of a standardized feeding regimen, antibiotic stewardship, or abnormal microbial colonization of the gut [37,38]. Indeed, the latter factors must be regarded as more likely causes. In the present study mortality was very low in both groups (HMF 5%, BMF 3%). The overall mortality in infants with a gestational age between 22–29 weeks in the VON is assumed to be 20% [35]. In the present study, the overall mortality in infants with a birthweight below 1000 g across the two study centers was also between 16–19% per year (data not shown), but one inclusion criterion was “initiation of HM fortification”. Therefore, only infants with a minimal enteral intake of 100 mL/kg were included, indicating that the sickest infants who had already died were not included.

### 4.4. Strengths and Limitations

This academic study focused on a large group of ELBW-infants at two neonatal tertiary care centers who received an exclusive human milk diet up to 32+0 weeks in a clinical setting. Growth velocity was determined and compared with previously validated, standardized methods. Furthermore, data on feeding tolerance at fortifier introduction and fortifier transition provided clinically relevant information on an exclusive milk diet. This was a retrospective study, which clearly has a potential for bias from various sources. Nevertheless 1:1 matching for birthweight (within 100 g) and gestational age at birth (within seven days) should have lowered potential bias.

While there was no difference in growth velocity from birth to 37+0 weeks between groups, the most sensitive endpoint showed a significant difference in favor of BMF.

## 5. Conclusions

In this study, growth velocity in ELBW infants was significantly lower in an HMF regimen from introduction of fortifier up to 32+0 weeks of gestation in comparison to a BMF regimen. This could be related to the composition of the HMF fortifier, as it has a high protein and fat proportion and a low carbohydrate proportion which might be unfavorable for ELBW-infants. The impaired ability for fat digestion and absorption might account for significantly lower growth velocity in the HMF group, since fat is the major caloric contributor in the HMF regimen. An exclusive human milk diet did not affect parameters of feeding tolerance, metabolism, morbidity or mortality. Overall, results from our study and those of others (Table 7) do not support the superiority of HMF over BMF.

## Figures and Tables

**Table 1:** ESPGHAN (European Society for Pediatric Gastroenterology, Hepatology and Nutrition) Recommdations [1] for macronutrient intake for infants with a birthweight <1500 g, macronutrient content of human milk (HM), HM + human milk based fortifier (HMF), and HM + bovine based fortifier (BMF).

**Macronutrient**	**ESPGHAN** **Recommendations**	**HM** **(100 mL)**	**HM + HMF** **(100 mL)**	**HM + BMF** **(100 mL)**
Energy (kcal)	110–135/kg/day	70	91	85.27
Protein (g)	4.0–4.5/kg/day	1.81	3.07	2.92
Fat (g)	4.8–6.6/kg/day	4.0	5.5	4.0
Carbohydrates (g)	-	6.95	7.63	9.69
**Macronutrient-Energy-Ratio**	**ESPGHAN** **Recommendations**	**HM** **(100 kcal)**	**HM + HMF** **(100 kcal)**	**HM + BMF** **(100 kcal)**
Protein-energy-ratio (g/100 kcal)	3.6–4.1	2.59	3.37	3.42
Carbohydrate-energy-ratio (g/100 kcal)	10.5–12.0	9.9	8.4	11.4
Lipid-energy-ratio (g/100kcal)	4.4–6.0	5.7	6.0	4.7

HM = human milk, HMF = human milk-based fortifier, BMF = bovine based human milk fortifier, References by ESPGHAN = European society of Pediatric Gastroenterology, Hepatology and Nutrition.

**Table 2:** Baseline characteristics of the study population.

Variable	HMF Group (*n* = 96)	BMF Group (*n* = 96)	*p*-Value
	**Median (IQR)**	**Median (IQR)**	
Gestational age (weeks + days)	26+1 (24+6, 27+1)	25+6 (24+5, 27+3)	n.s.
Birth weight (g)	752 (659, 893)	773 (650, 890)	n.s.
Birth weight (zscore)	−0.3 (−1.1, 0.3)	−0.4 (−1.2, 0.2)	n.s.
Birth length (cm)	32.8 (30.9, 34.5)	33.0 (31.0, 35.0)	n.s.
Birth length (zscore)	−0.6 (−1.4, 0.0)	−0.5 (−1.4, 0.1)	n.s.
Birth head circumference (cm)	23.5 (22.3, 24.5)	23.5 (22.5, 24.5)	n.s.
Birth head circumference (zscore)	−0.3 (−1.1, 0.3)	−0.2 (−1.1, 0.3)	n.s.
Apgar-Score 5 min	8 (8, 9)	8 (8, 9)	n.s.
Apgar-Score 10 min	9 (9, 9)	9 (9, 9)	n.s.
Arterial umbilical cord pH	7.3 (7.2, 7.3)	7.3 (7.2, 7.4)	n.s.
	***n* (%)**	***n* (%)**	
Small for gestational age	21 (22%)	15 (16%)	n.s.
Male	45 (47%)	42 (44%)	n.s.
Multiple births	24 (25%)	36 (38%)	n.s.
Sectio caesearea	80 (83%)	82 (85%)	n.s.
Lung maturation	89 (93%)	90 (94%)	n.s.
Lung maturation complete	71 (74%)	69 (72%)	n.s.
Premature rupture of membranes	38 (40%)	22 (23%)	<0.05
Preeclampsia/Eclampsia	11 (11%)	9 (9%)	n.s.

HM = human milk, HMF = human milk-based fortifier, BMF = bovine based human milk fortifier, IQR = interquartile range; Mann-Whitney U test, Chi-Squared test (or Fishers Exact test) as appropriate.

**Table 3:** Growth velocities and anthropometry of the study population.

Variable	HMF Group (*n* = 96)	BMF Group (*n* = 96)	*p*-Value
	Median (IQR)	Median (IQR)	
**Hierarchical structure of endpoints**			
Growth velocity (g/kg/d) birth to 37+0 weeks	15.2 (14.2, 16.7)	15.6 (13.9, 16.7)	n.s.
Growth velocity (g/kg/d) fortifier introduction to 32+0 weeks	16.5 (14.5, 19.1)	18.9 (15.3, 21.3)	0.009
Growth velocity (g/kg/d) birth to fortifier introduction	13.5 (7.4, 16.8)	10.7 (4.8, 15.3)	0.09
Growth velocity (g/kg/d) birth to 32+0 weeks	15.3 (14.0, 17.7)	15.1 (13.6, 17.0)	n.s.
Growth velocity (g/kg/d) 32+0 to 37+0 weeks	15.5 (13.2, 17.2)	15.9 (13.9, 17.7)	n.s.
**32+0 weeks of gestational age**			
weight (g)	1442 (1267, 1580)	1430 (1240, 1598)	n.s.
weight (zscore)	−1.0 (−1.5, −0.7)	−1.1 (−1.5, −0.6)	n.s.
length increase (cm/week)	1.0 (0.7, 1.3)	0.9 (0.6, 1.3)	n.s.
length (cm)	38.0 (36.0, 39.4)	38.0 (36.7, 39.5)	n.s.
length (zscore)	−2.1 (−2.9, −1.5)	−2.1 (−2.6, −1.5)	n.s.
head circumference increase (cm/week)	0.6 (0.4, 0.8)	0.6 (0.4, 0.8)	n.s.
head circumference (cm)	26.5 (25.3, 27.5)	26.6 (25.5, 27.5)	n.s.
head circumference (zscore)	−2.0 (−2.8, −1.5)	−2.0 (−2.0, −1.4)	n.s.
**37+0 weeks of gestational age**			
weight (g)	2500 (2136, 2722)	2427 (2155, 2722)	n.s.
weight (zscore)	−1.0 (−1.8, −0.6)	−1.2 (−1.8, −0.6)	n.s.
length gain (cm/week)	1.1 (1.0, 1.3)	1.1 (0.9, 1.2)	n.s.
length (cm)	44.0 (43.0, 45.5)	44.5 (43.0, 46.0)	n.s.
length (zscore)	−1.9 (−2.3, −1.2)	−1.7 (−2.3, −1.0)	n.s.
head circumference gain (cm/week)	0.8 (0.7, 0.9)	0.8 (0.7, 0.8)	n.s.
head circumference (cm)	31.2 (30.0, 32.0)	31.5 (30.5, 32.5)	n.s.
head circumference (zscore)	−1.3 (−2.0, −0.9)	−1.2 (−1.7, −0.6)	n.s.

HM = human milk, HMF = human milk based fortifier, BMF = bovine based human milk fortifier; Mann-Whitney U test.

**Table 4:** Parameters of parenteral and enteral nutrition.

Variable	HMF Group (*n* = 96)	BMF Group (*n* = 96)	*p*-Value
	**Median (IQR)**	**Median (IQR)**	
Time to full enteral feedings (days)	26 (19, 38)	20 (14, 32)	0.0018
Parenteral Nutrition (days)	28 (19, 44)	22 (15, 40)	0.01
Central line (days)	30 (20, 49)	24 (15, 45)	0.01
Fortifier days up to 32+0 weeks	20 (8, 28)	19 (10, 30)	n.s.
Fortifier days up to 37+0 weeks	48 (35, 59)	45 (31, 57)	n.s.
	***n* (%)**	***n* (%)**	***p*-value**
Enteral nutrition up to 32+0 weeks			
- HM + Fortifier	84 (88%)	89 (93%)	n.s.
- deceased	3 (3%)	3 (3%)	n.s.
Enteral nutrition at 37+0 weeks			
- HM	5 (5%)	0 (0%)	n.s.
- HM + BMF	39 (41%)	38 (39.5%)	n.s.
- Formula	36 (38%)	37 (38.5%)	n.s.
- Mixed	13 (14%)	18 (19%)	n.s.

HM = human milk, HMF = human milk based fortifier, BMF = bovine based human milk fortifier, IQR = interquartile range; Mann-Whitney U test, Chi-Squared test (or Fishers Exact test) as appropriate.

**Table 5:** Feeding tolerance at fortifier introduction: comparison of data from the present study with the literature.

		HMF Group *n* (%)	BMF Group *n* (%)	*p*-Value
Present study *^1^	Feeding intolerance	8 (8%)	5 (5%)	n.s.
	Gastric residuals	6 (6%)	3 (3%)	n.s.
	Interruption	22 (21%)	18 (19%)	n.s.
O’Connor et al. [27] *^2^	Feeding intolerance	9 (14.4%)	0 (0%)	n.s.
	Gastric residuals	26 (40.6%)	25 (41.1%)	n.s.
	Interruption	17 (26.6%)	20 (32.8%)	n.s.

*^1^ Feeding intolerance and gastric residuals for the first 3 days of introduction, Interruption from introduction until 32+0, *^2^ whole fortification period; HM = human milk, HMF = human milk based fortifier, BMF = bovine based human milk fortifier.

**Table 6:** Morbidity and Mortality of the study population.

Variable	HMF Group (*n* = 96) *n* (%)	BMF Group (*n* = 96) *n* (%)	*p*-Value
Mortality	5 (5%)	3 (3%)	n.s.
IVH	26 (27%)	23 (24%)	n.s.
- I and II	13 (14%)	15 (16%)	
- III and IV	13 (14%)	8 (8%)	
PVL	3 (4%)	1 (1%)	n.s.
Sepsis	45 (47%)	41 (43%)	n.s.
PDA	58 (60%)	56 (58%)	n.s.
- Ligature	4 (4%)	5 (5%)	
BPD	29 (30%)	23 (23%)	n.s.
ROP	53 (55%)	45 (47%)	n.s.
- I-III	53 (55%)	43 (45%)	
- III+	0 (0%)	2 (2%)	
NEC	10 (10%)	8 (8%)	n.s.
- Abdominal surgery	7 (7%)	7 (7%)	n.s.
FIP	5 (5%)	5 (5%)	n.s.

IVH = intraventricular hemorrhage, PVL = periventricular leukomalacia, PDA = patent ductus arteriosus, BPD = bronchopulmonary dysplasia, ROP = retinopathy of prematurity, NEC = necrotizing enterocolitis, FIP = focal intestinal perforation, HM = human milk, HMF = human milk based fortifier, BMF = bovine based human milk fortifier; Chi-Squared test (or Fishers Exact test) as appropriate.

**Table 7:** Growth velocities (GV)—comparison between data from the present study with the literature.

Study	HMF Group	BMF Group	*p*- Value	Method	Timeframe
**Present study**	*n* = 96	*n* = 96	n.s.	Patel	Birth to 37+0
BW, median (IQR)	752 (659, 893)	773 (650, 890)			
GV, median (IQR)	15.2 (14.2, 16.7)	15.6 (13.9, 16.7)			
**Sullivan et al. [11]**	*n* = 67	*n* = 69	n.s.	Daily weights	*1
BW, mean (SD)	14.2 (11.9, 15.8)	15.1 (12.8, 17.0)			
GV, median (IQR)	945 (± 202)	922 (± 197)			
**Colacci et al. [26]**	*n* = 39	*n* = 46	n.s.	Patel	Birth to discharge
BW, mean (SD)	783 (± 143)	770 (± 137)			
GV, mean (SD)	13.1 (± 4.0)	12.1 (± 5.2)			
**Hair et al. 2013 [29]**	*n* = 104	-	-	Daily weights	Birth to discharge
BW, mean (SD)	913 (±182)				
GV, mean (SD)	24.8 (± 5.4)				
Huston et al. [16] *^2^	*n* = 94	*n* = 111	-	Patel	Birth to discharge
BW, mean (SD)	904 (± 200)	959 (±174)			
GV, mean (SD)	13.6 (± 1.6)	14.1 (± 1.8)			
**Assad et al. [12]**	*n* = 87	*n* = 127	n.s.	Two-point average	Birth to discharge
BW, range	490–1700 g	490–1700 g			
GV, mean (SD)	11.6 (± 2.7)	11.6 (± 2.5)			

^*1^) birth to earliest of the following milestones: 91 days of age, discharge from hospital, or attainment of 50% oral feedings (i.e., four complete oral feedings per day), *2) birth until 36 weeks or weaned from fortifier (whichever occurred first), transferred to a nonstudy institution, removed from the study, or death; HM = human milk, HMF = human milk based fortifier, BMF = bovine based human milk fortifier, GV = growth velocity, BW = birthweight, IQR = interquartile range, SD = Standard Deviation.
